# The Structure of the Human Placenta

**Published:** 1842-05

**Authors:** John Dalrymple

**Affiliations:** Assistant Surgeon to the London Ophthalmic Infirmary


*The Structure of the Human Placenta.* By John Dalrymple, Esq., Assistant Surgeon to the London Ophthalmic Infirmary.—[In the early part of last year, Mr. D., having pursued some anatomical investigations of the structure of the human placenta at the term, and having made several drawings of the injected capillaries of the tuft, afterwards had an opportunity of seeing the copies of Weber's drawings, given in the Icones Physiologicæ of Wagner, and transferred to the pages of Dr. Willis's translation of the latter author's Physiology.

The resemblance of the present drawings to those given by Dr. Willis was so striking as to go far, of itself, to prove the correctness of both draughtsmen, and to corroborate the views entertained by Weber of the anatomical conditions of the organ. They differ, also, from the engravings of Dr. Reid (given in the January number of the Edinburgh Medical and Surgical Journal for 1841), inasmuch as no where could be seen an artery and vein running side by side, forming an apparent single vessel, though with a double tube, and terminating abruptly in blunt extremities, where the anastomoses took place between them.

The following are the results of Mr. D's. researches and examinations as given in a recent number of the London Medical Gazette:—]

1. That the placenta was made up of innumerable subdivisions of the umbilical vessels, terminating in beautiful coils and convoluted capillaries, which formed tufts or bouquets of vessels clothed by a prolongation of the endo-chorion derived from the fœtal surface of the organ.

2. That nowhere did a division of an umbilical artery terminate otherwise than in a branch of the umbilical vein; and each branch, as well as tuft of vessels, was covered by a prolongation of the before named membrane.

3. That each tuft was, in fact, a real villus; the endo-chorion being covered externally with an epithelium-like tissue, having nucleated cells and corpuscles.

4. The uterine surface of the placenta is covered by the decidua which does not appear to enter further into the structure of the organ than between the lobules; and the depth to which it thus penetrates varies with the depth of the fissures.

5. That fibrous bands stretch from the fœtal to the placental surface of the organ, giving firmness and support to the vessels.

6. That there are no defined cells in the placenta, but that the nutrient fluids of the mother are poured into the interstices of the tufts, which are not bound or connected together by a common cellular tissue.

7. That on the decidual surface of the placenta are thinly scattered, here and there, blunt conical papillæ, about a line and a half in length, made up of innumerable coiled and contorted capillaries. Query.—Are these the analogues of the fœtal cotyledons of the ruminant?

From these observations, which were given in a minute detail, the author has attempted to simplify the functions of the human placenta.

He observes, that in the incubated egg, in consequence of the non-connexion between the embryo and parent, a nutrient and a respiratory organ is indispensable, and hence the more complicated system of vessels. That in the oviparous vertebrata, the vitellary sac, aud the omphalo-mesenteric vessels represent the placenta of mammalia, which is the absorbent organ of the fœtus. But while in the one case the nutrient materials of the mother, already aerated by her lungs, are conveyed by the uterine arteries for absorption and nutrition of the embryo, in the other the materials of the blood are absorbed by the folds of the vitelline sac, and conveyed through the circulation of the young bird, requiring, however, contact with oxygen for a second circulation: hence a new membrane, or one that is persistent up to the time of independent respiration, viz: the allantois; and hence, also, the more complicated system of its vessels. The allantois, as a respiratory membrane, exists only as a rudimentary organ in mammalia, and the function of the placenta being solely that of nutrition by already oxygenized materials, the cord contains only a simple system of incurrent and excurrent vessels.

